# The Effect of Bacterial Endotoxin LPS on Serotonergic Modulation of Glutamatergic Synaptic Transmission

**DOI:** 10.3390/biology9080210

**Published:** 2020-08-06

**Authors:** Jate Bernard, Abigail Greenhalgh, Oscar Istas, Nicole T. Marguerite, Robin L. Cooper

**Affiliations:** Department of Biology, Center for Muscle Biology, University of Kentucky, Lexington, KY 40506-0225, USA; jatebernard@gmail.com (J.B.); Abigail.Greenhalgh@uky.edu (A.G.); owis222@g.uky.edu (O.I.); Nicole.Marguerite@uky.edu (N.T.M.)

**Keywords:** lipopolysaccharide, endotoxin, *Serratia marcescens*, neuromodulation, serotonin, neuromuscular junction

## Abstract

The release of the endotoxin lipopolysaccharides (LPS) from gram-negative bacteria is key in the induction of the downstream cytokine release from cells targeting cells throughout the body. However, LPS itself has direct effects on cellular activity and can alter synaptic transmission. Animals experiencing septicemia are generally in a critical state and are often treated with various pharmacological agents. Since antidepressants related to the serotonergic system have been shown to have a positive outcome for septicemic conditions impacting the central nervous system, the actions of serotonin (5-HT) on neurons also exposed to LPS were investigated. At the model glutamatergic synapse of the crayfish neuromuscular junction (NMJ), 5-HT primarily acts through a 5-HT2A receptor subtype to enhance transmission to the motor neurons. LPS from *Serratia marcescens* also enhances transmission at the crayfish NMJ but by a currently unknown mechanism. LPS at 100 µg/mL had no significant effect on transmission or on altering the response to 5-HT. LPS at 500 µg/mL increased the amplitude of the evoked synaptic excitatory junction potential, and 5-HT in combination with 500 µg/mL LPS continued to promote enhanced transmission. The preparations maintained responsiveness to serotonin in the presence of low or high concentrations of LPS.

## 1. Introduction

Septicemia induced by gram-negative bacteria is life threatening and can trigger a whole-body immune response which can further pathological conditions with elevated levels of released cytokines. Therapeutic treatments of bacterial septicemia vary depending on many conditions of the infection and health status of the person or animal [1]. There is a heavy focus on reducing the effects of the cytokines on target tissue [2,3]. Blocking the effect of LPS is approached by focusing on the receptors for LPS, although such therapeutics have not been forthcoming.

Along with attempting to target receptors for LPS, there are also many therapeutic approaches to reducing the immune responses [1]. To understand the treatments, one can approach this task from a whole animal or a cellular level using various animal models. Microglia in the nervous system are major contributors to secondary cytokine release. Findings suggest antidepressants such as serotonin reuptake inhibitors (SSRIs: fluoxetine, sertraline etc.) show some therapeutic effectiveness in anti-inflammatory properties on microglia [4]. Considering that multi-organ dysfunction can occur with septicemia, the normal excretion of prescribed pharmacological compounds can be altered, thus altering the levels within the body. It is known that LPS modifies cellular membrane potential and synaptic transmission, [5] as well as many types of therapeutics either directly or indirectly. Therefore, it is of interest to understand how the targets of medications may be influenced by the direct compounding action of LPS. Medications to manage depression and anxiety commonly involve the serotoninergic system on either the reuptake transporters (i.e, SSRIs) or the specific serotonin (5-hydroxytryptamine or 5-HT) receptors [1]. The serotonin receptor subtype 5-HT2A is one of the receptor subtypes targeted due to the relationship with depression and altering mood [6,7]. Serotonin as well as agonists to 5-HT2A increase cellular activity and synaptic responses [8,9].

Serotonin modulates not only the CNS but also peripheral nerves and tissues, which LPS could also have access to during septicemia [10,11]. It has been shown that LPS can cross the blood–brain barrier (BBB) if the BBB is compromised by prolonged septicemia and cytokine-induced inflammation. LPS systemic injection can even induce the breakdown of the BBB [12]. Thus, LPS can then have a direct influence on central neurons and microglia as well as sites where serotonin would have action.

In mammals, LPS binds to Toll-like receptor 4 (TLR4) known as the CD14/TLR4/MD2 receptor complex [13,14]. The TLR4 receptor is conserved from *Drosophila* to primates [15]. In fact, the Toll receptors were first discovered in *Drosophila melanogaster*, which led to the discovery of these receptors for LPS in mammals. The arthropod cousin of insects are crustaceans, and like insects, they use an innate immune system to protect from foreign substances such as bacteria when they enter the body. The innate immune system promotes rapid aggregation of the bacteria and related endotoxins [16]. The blood (i.e., hemolymph) of the horseshoe crab has been used for many years to test for the presence of gram-negative bacteria related to human healthcare using the Limulus amebocyte lysate assay via this innate immune response [17,18,19].

Since the strain of gram-negative bacteria from *Serratia marcescens* is commonly associated with septicemia in invertebrates and humans [20,21,22,23,24], the LPS of this bacterial strain has recently been used to address its direct action on neuronal function and muscle physiology [5]. It was shown that LPS from *Serratia marcescens* increases the synaptic efficiency of the glutamatergic synapses at the crayfish neuromuscular junction (NMJ). It appears the mechanisms of action increasing the amplitude of evoked excitatory junction potentials are presynaptic, enhancing vesicle fusion [5,25,26]. 5-HT also enhances the amplitude of evoked excitatory junction potentials at the crayfish NMJ by increasing the evoked vesicle fusion [27,28,29,30]. This effect has been established to be induced by a 5-HT2A-like receptor, which mediates a phospholipase C (PLC) activation and a subsequent IP3 and DAG cascade [31,32,33]. Thus, this study examined if the increased synaptic responses at the crayfish NMJ induced by exposure to LPS could be further enhanced or altered by the addition of 5-HT. We hypothesized that the mechanism of action of LPS would severely dampen the response to serotonin as the potential mechanisms of cellular responses may merge in their ability to enhance synaptic transmission.

## 2. Materials and Methods

### 2.1. Animals and Dissection

Experiments were performed using Red Swamp Crayfish (*Procambarus clarkii).* They were obtained from a distribution center in Atlanta, GA, USA and delivered to a local supermarket in Lexington, KY, USA, where they were purchased. Throughout the study, midsized crayfish measuring 6–10 cm in body length were used. Each animal was housed in individual standardized plastic containers with weekly exchanged dry fish food and aerated water (20–21 °C).

**Figure 1:**
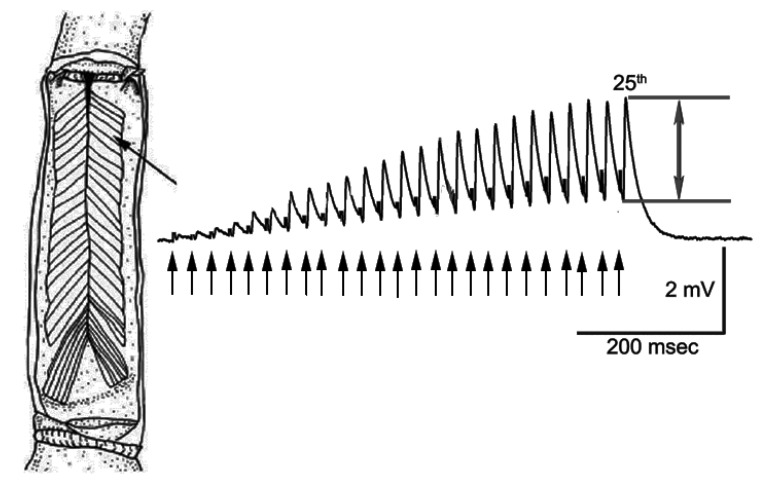
The opener muscle in the 1st walking leg of a crayfish is innervated by a single excitatory motor neuron. The distal muscle bundles provide facilitated responses due to a train of stimuli. A response to a 40 Hz train of 25 stimuli is shown. The amplitude of the 25th response was used as a measure (as shown between the two arrows). The closed arrow heads indicate the 25 stimulus pulses also shown as stimulus artifacts in the response trace.

The opener muscle of the first walking legs of the crayfish was used. This preparation of crayfish has been historically reviewed for studying synaptic transmission [34]. The most distal muscle fibers in the preparation were used as these fibers provide for consistency among preparations [35] (Figure 1). The tonic phenotype of the innervation of these fibers is low output and fatigue resistant but has pronounced facilitation [36,37]. The dissection to expose and selectively stimulate the excitatory motor neuron of the opener muscle is described in textual and video format [34]. The excitatory neuron is isolated from the inhibitor neuron and stimulated in the meropodite segment. 

### 2.2. Physiological Recordings

The stimulation paradigm was a train of 25 pulses at 40 Hz with 20 s between trains. The amplitude of the 25th EJPs was used as an index for the effects of LPS and 5-HT as shown in Figure 1.

Suction electrodes made from plastic tips were used to stimulate the cut nerves (details of making the suction electrodes are provided in Baierlein et al. [38]). The nerve was stimulated with supramaximal pulses using a suction electrode and Grass S-88 stimulator to elicit EJPs. Intracellular recordings were made with sharp glass electrodes filled with 3 M KCl at 40 megaOhm resistance. Responses were recorded with an AxoClamp 2B (Molecular Devices, LLC., San Jose, CA, USA), converted with a PowerLab, 4SP (ADInstruments, Colorado Springs, CO, USA), and analyzed with LabChart 7.0 (ADInstruments, Colorado Springs, CO, USA) which were recorded on a computer at a 20 kHz sampling rate.

Dissected preparations were maintained in crayfish saline, a modified Van Harreveld’s solution (in mmol/ L: 205 NaCl; 5.3 KCl; 13.5 CaCl_2_·2H_2_O; 2.45 MgCl_2_·6H_2_O; 10 glucose; 0.5 HEPES adjusted to pH 7.4). Commercial LPS from *Serratia marcescens* was dissolved in physiological saline the day of experimentation. This LPS may also contain some associated peptidoglycans from *S. marcescens.* A concentration of 100 and 500 µg/mL of LPS was used to compare to earlier studies used at the crayfish and *Drosophila melanogaster* NMJs. Also, 5-HT was prepared from a frozen stock solution of 1 mM to 1 µM on the day of experimentation. Stock 5-HT was prepared in small aliquots and protected from light exposure. All chemicals for the saline, LPS, and 5-HT were obtained from Sigma-Aldrich (St. Louis, MO, USA).

The non-linear summation of the amplitude evoked EJP using Martin’s correction factor is an approach to correct for the driving potential changing as the amplitude of the EJP becomes closer to the ionic drive for the potential [39]. The Martin correction factor appears to have a substantial alteration in the estimated potential due to the driving gradient for EJPs greater than 15% of the resting membrane potential, [39] but since this was not the case for the 25th EJP in these studies it was not necessary to use the correction factor.

LPS exposure was obtained by exchanging the untainted saline with LPS containing saline while maintaining intracellular recording. The high concentration of LPS was also used to compare with previous studies using the muscles of frog and crayfish and the CNS of rodents [5,40]. The LD50 in rodents for LPS from *S. marcescens* is 650 µg/mL (6 × 10^6^ CFU-colony-forming units [41]). This was another reason to use a relatively high concentration for crayfish, since they are likely exposed to high levels of gram-negative bacterial strains in their native environment.

### 2.3. Statistics

All data are expressed as an average value along with the standard error of the mean (i.e., ±SEM) or as a percent change. The rank-sum pairwise test or a sign test was used to compare the differences in responses before and after exchanging solutions. An ANOVA analysis is used to compare among data sets of varying experimental conditions. The analysis was performed with Sigma Stat software. *p* of ≤ 0.05 is considered as statistically significant.

## 3. Results

The stimulation paradigm consisted of providing a train of 25 pulses at 40 Hz with 20 s between trains. The amplitude of the 25th EJPs within the response train was used as an index for the effects of LPS and 5-HT (Figure 1). The rationale for using 25th pulses in a train is that, by the 25th pulse, a plateau in the amplitude of the EJP is usually reached at room temperature for the opener NMJs [42].

**Figure 2:**
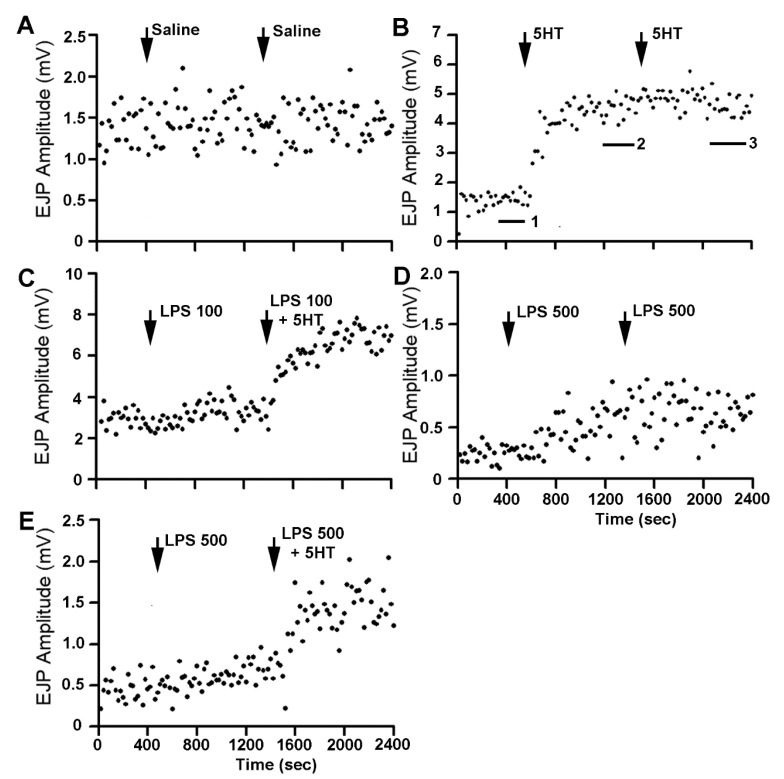
Representative responses in the amplitude of the 25th EJP with varying experimental paradigms. (**A**) The control conditions with exchanging of the bathing solution and time of recording for the saline to be exchanged with another saline. (**B**) The bathing saline exchanged with one containing 5-HT (1 µM) and again exchanged with saline containing 5-HT (1 µM). Note that no further increases in the amplitude of the EJP with the 2nd exchange occurred. In this trace, three bars are shown (1, 2 and 3). These are the representative time windows used for obtaining averages in all the files (200 s in saline before exchanging the bath as the control reference; 200 s before the 2nd bath exchange and lastly 200 s after 800 s of incubation in the 2nd bath exchange). (**C**) The effect of LPS at 100 µg/mL by itself and then with LPS 100 µg/mL combined with 5-HT (1 µM). (**D**) The effect of LPS at 500 µg/mL by itself and exchanged a second time with LPS at the same concentration of 500 µg/mL. (**E**) The effect of LPS at 500 µg/mL by itself and exchanged to LPS (500 µg/mL) combined with 5-HT (1 µM). Generally, each preparation was bathed for 400 s in saline before being exchanged with the next bathing solution in which the preparations were bathed in for 1000 s before being exchanged into the last solution for another 1000 s. The arrows indicate the approximate times of exchanging of the bath for these representative preparations.

**Figure 3:**
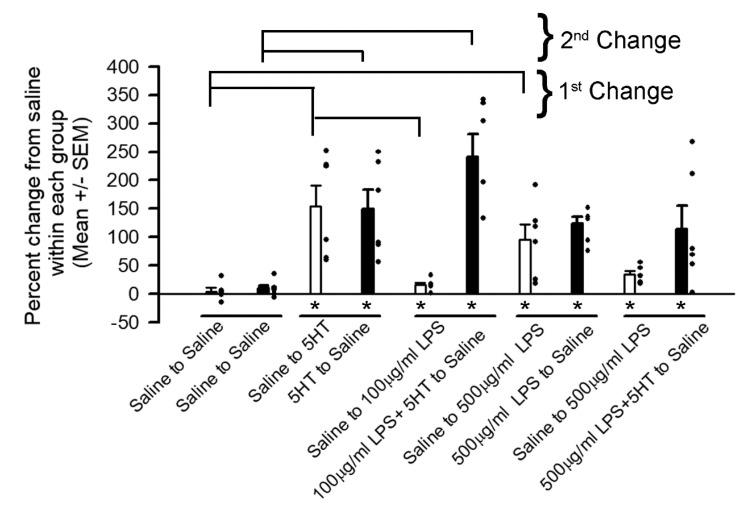
The percent change in the 25th EJP amplitude for each condition with respect to the initial saline exposure. The dots represent the percent change for each preparation under each condition (*n* = 6 for each). The histograms are the mean (+/−SEM) for the percent change with respect to the initial saline exposure. The first bar (open) in each paradigm is the percent change of the saline for the first bath exchange and the second bar (closed) is the percent change after the last bath exchange with respect to the initial saline exposure. The average amplitudes in the EJP over the time frames are depicted in Figure 2B as 1, 2 and 3. The * under the bar indicates a significant effect (*p* < 0.05, Sign Test, non-parametric) on the initial values in saline for the given paradigm. The bars above the histograms represent significant changes for either the effect of the 1st bath change on the saline-only control paradigm or the effect of the 2nd bath exchange on the 2nd saline control paradigm. Thus, the saline-saline-saline result is given.

The action of 5-HT was robust in increasing the amplitudes of the EJPs with or without the presence of LPS. For examining the effect of changing the bathing solution as well as the prolonged time of the recordings, controls were run with only saline and exchanges with saline (Figure 2A). Note the mean in the amplitude of the EJP in the 200 s (20 samples) was used to index for the time in saline before the first bath exchange as well as the time before the second bath exchange. See Figure 2B for an illustration of the times the averages were obtained by the labels 1, 2, and 3 in the figure. Exposure to 5-HT (1 µM) rapidly increased the amplitude (*p* < 0.5, Paired *t*-Test; *n* = 6), and exchanging the bathing media with the same concentration of 5-HT did not further increase the amplitudes (Figure 2B). The first exposure of 5-HT likely produced a saturation of the receptors or of a downstream cellular cascade since the response appeared to plateau. Exposure to LPS at 100 µg/mL did result in an increase in EJP amplitude for each preparation examined (*p* < 0.5, Paired *t*-Test; *n* = 6 and Sign test non-parametric) but only resulted in a slight average increase (Figure 3) and was not significantly different than controls. The combination of LPS at 100 µg/mL and 5-HT after initial exposure to LPS resulted in a rapid rise in the amplitude of the EJP. Additionally, exposure to LPS at 500 µg/mL had a larger effect of increasing the EJP amplitude than exposure to LPS at 100 µg/mL (Figure 2C; *p* < 0.5, Paired *t*-Test; *n* = 6 and Sign test non-parametric) and to saline controls. The second exposure to the same concentration continued to increase the amplitude of the EJP, but without a significant effect from the first exposure of LPS at the same concentration (Figure 2D). However, exposure to 5-HT with LPS (500 µg/mL) after exposure to LPS at 500 µg/mL resulted in a rapid increase in the amplitude of the EJPs (Figure 2E; *p* < 0.5, Paired *t*-Test; *n* = 6 and Sign test non-parametric Figure 3). There was no significant difference in the initial amplitudes in the starting saline conditions among the different paradigms.

The percent change within each preparation from the initial saline was determined as an index to normalize comparisons among preparations as each had varying amplitudes in the initial EJPs in saline (Figure 3). The means (+/−SEM) in the percent changes of the groups were also determined. Non-parametric analyses (Sign test) were determined for the percent change in each paradigm for the first bath exchanged and for the second bath exchange to that of the initial saline exposure. Note the asterisk under the bar graphs for the groups are comparisons within individual groups. A Kruskal-Wallis one-way analysis of variance on ranks was used to compare among groups for the first bath exchange and separately for the second bath exchange to the initial amplitudes of EJP in saline (as shown in bars above the histograms). The open histogram bars represent the first bath exchange and the filled histogram bars represent the second bath exchange.

The paradigm for the first bath change is compared to the first bath changes in the other paradigms and the second bath exchanges are compared to the second bath exchange in saline alone.

**Figure 4:**
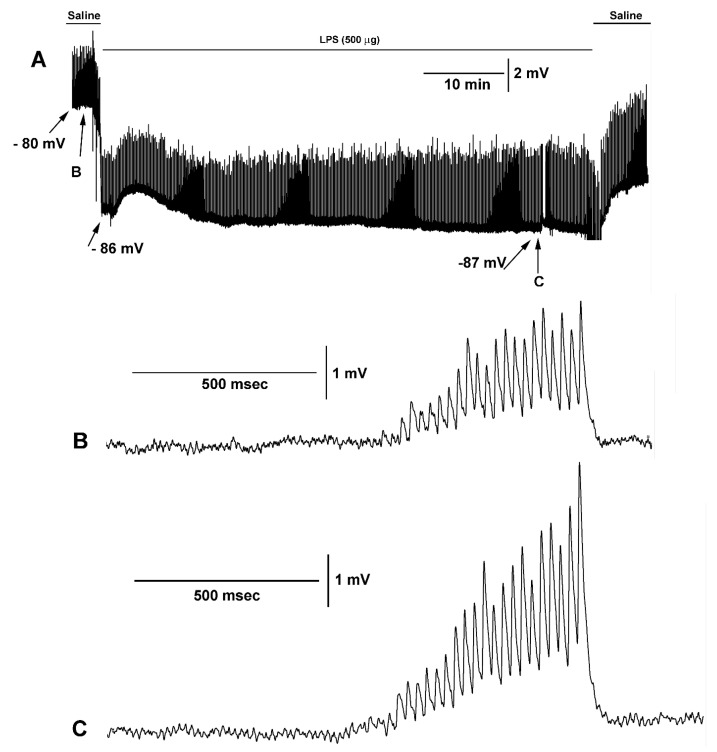
The effect of LPS on membrane potential and evoked synaptic transmission. (**A**) A prolonged exposure to LPS for 1 h while evoking trains of 25 stimuli at 40 Hz every 10 s. Note the rapid hyperpolarization upon exposure of LPS and gradual rise in the amplitudes of the EJPs. The membrane potentials are listed at various time points. (**B**) An enlarged view of one stimulus train of 25 pulses at 40 Hz prior to LPS exposure. (**C**) An enlarged view of one stimulus train of 25 pulses at 40 Hz after 55 min to LPS exposure. Note the EJPs need to be facilitated within single stimulus trains to be observed.

The hyperpolarization of the muscle is rapid upon exposure to LPS while the amplitude of the EJPs gradually increases in amplitude. A representative example is shown in Figure 4A for an hour of exposure of LPS while stimulating the motor nerve every 10 s with 25 stimuli pulses at 40 Hz. The membrane potential changes rapidly upon LPS and trends to recover upon removal of LPS. The rapid changes in membrane potentials are also observed in larval *Drosophila* muscles [5]. The increase in the amplitude of the EJPs is more gradual as compared to the rapid membrane potential changes. Enlarged views of the responses due to the stimulus train before (Figure 4B) and during LPS exposure (Figure 4C) are illustrated.

## 4. Discussion

The direct actions of LPS on neurons present an active research area since the effects are not well understood independently from the secondary effects of cytokines released from multiple cell types upon LPS exposure [3]. Thus, the secondary effects of cytokines make it challenging to determine the interaction of LPS and neuromodulators during synaptic transmission. The isolated NMJ of the crayfish, bathed in a known minimal physiological saline of salts, allows an assessment of LPS and 5-HT that is independent of the whole animal and the circulatory system, as high concentrations of LPS injected into the circulation can kill crayfish [25].

Since the opener NMJ preparation is physiologically stable for hours with relatively low frequency stimulation or short stimulus trains with prolonged intervals of rest, there was little depression in synaptic efficacy [9,42,43,44]. Also, the effects of LPS exposure over prolonged periods of time were previously addressed in this preparation [26]. LPS at 500 µg/mL of *Serratia marcescens* promoted a gradual increase in the EJP amplitude after 20 to 30 min; however, the effect of muscle hyperpolarization was within 1 to 2 min of LPS exposure. In the study herein, after 30 min of LPS exposure, there was an increase in the amplitude of the EJP observed for the 500 µg/mL but not for the 100 µg/mL. The rapid increase in the EJP amplitude with subsequent exposure of 5-HT and LPS illustrated that a relatively high concentration of LPS did not block the effect of 5-HT, nor was the high concentration of LPS toxic to the synaptic preparation where it could not respond to the modulation by 5-HT.

The effect of 5-HT with prior exposure to LPS at 100 µg/mL or 500 µg/mL was not significantly different, which further supports the notion that LPS does not substantially alter the responsiveness of the preparation to 5-HT. In addition, a second exposure to a high concentration of LPS did not result in a further hyperpolarization of the muscle fiber or a rapid alteration in the amplitude of the EJP, indicating that potentially a maximal effect of LPS was obtained with the first exposure. The mechanism by which LPS promotes synaptic transmission is likely presynaptic via promoting vesicular fusion. Since the membrane potential of the muscle rapidly hyperpolarizes while the amplitude of the evoked EJPs takes longer to occur, the increased amplitude is not due to an increased driving gradient on the muscle through the ionotropic glutamate receptors. This supports a presynaptic effect since the evoked amplitude is a more synchronized occurrence of vesicle fusion and there are no changes in the amplitude of spontaneous events during LPS exposure on the crayfish opener muscle [25]. However, 5-HT (100nM) at the crayfish NMJ increased the evoked responses by an IP3 induced secondary messenger cascade to promote vesicle docking as well as increase the occurrence of spontaneous quantal events [30,33,45]. Thus, it appears that LPS may alter Ca^2+^ dynamics with a slight increase in intracellular levels to increase residual calcium but not enough to increase the frequency of spontaneous events. It is possible that LPS alters a calcium pump or the sodium-calcium exchanger, which would lead to an increase in the evoked vesicle fusion. This might help to explain the pre-synaptic actions; however, the post-synaptic effect in the slight hyperpolarization is not well understood. The postsynaptic effect is even more prominent for the muscles of larval *Drosophila* at the same concentration of LPS [5,46]. The hyperpolarization does not appear to be due to calcium-activated potassium channels, activated nitric oxide synthase (NOS), or the opening of Cl-channels as some reports have proposed for the mechanism by which LPS alters membrane potential [5]. One speculative possibility is that activation of the Na-K ATPase pump transiently causes a hyperpolarization phase.

Since 5-HT action is mediated primarily by a 5-HT2A-like receptor subtype on the presynaptic terminals of crayfish NMJ [9,45], it may be feasible that LPS is also working in a similar secondary cascade but not likely by activating the 5-HT receptor since the application of 5-HT in the presence of LPS showed a normal response to 5-HT. To further address such a possibility, potentially blocking the PLC, IP3, and DAG secondary messenger cascades and examining the effect of LPS would help elucidate this mechanism [32,33,47,48]. We have recently approached this with *Drosophila* NMJ preparations as the larval *Drosophila* muscle hyperpolarize in a larger extent than the crayfish muscle for the same concentration of LPS [49]. We used RNAi knockdowns of potential receptors for LPS and the effects were no different from the application of LPS [5]. Note there are no known antagonists for any potential LPS receptor. Future experimentation in addressing the effects of LPS during 5-HT exposure would be beneficial to explore if further enhancement of synaptic transmission is possible. In addition, to address specifically presynaptic actions by LPS, focal macro-patch recordings over identified synaptic varicosities would allow for defined quantal measures of evoked responses [35]. Two electrode voltage clamping of the muscle fiber while examining evoked synaptic EJPs would also help to differentiate pre-and post-synaptic effects of LPS. 

## 5. Conclusions

High concentration of lipopolysaccharide enhances glutamatergic transmission at the crayfish neuromuscular junction. There is a rapid small hyperpolarization of the muscle fiber followed by a gradual enhancement in evoked synaptic excitatory potential. The increase in the evoked EJP appears to be due to presynaptic effects and does not compete with the mechanisms by which 5-HT enhances synaptic responses at this synaptic model. The 5-HT2A receptor mediated a cellular response, and the direct action of LPS likely enhances transmission by differing mechanisms
